# The impact of gardening on well-being, mental health, and quality of life: an umbrella review and meta-analysis

**DOI:** 10.1186/s13643-024-02457-9

**Published:** 2024-01-29

**Authors:** I. Panțiru, A. Ronaldson, N. Sima, A. Dregan, R. Sima

**Affiliations:** 1 Department of Horticulture and Landscape, University of Agricultural Sciences and Veterinary Medicine of Cluj-Napoca, Cluj-Napoca-Napoca, Romania; 2 Centre for Implementation Science, Health Service and Population Research Department, Institute of Psychiatry, Psychology and Neuroscience (IoPPN), King’s College London, London, UK; 3 Department of Technological Sciences, University of Agricultural Sciences and Veterinary Medicine of Cluj-Napoca, Cluj-Napoca-Napoca, Romania; 4 Department of Psychological Medicine, Institute of Psychiatry, Psychology and Neuroscience (IoPPN), King’s College London, London, UK

**Keywords:** Gardening, Horticultural therapy, Mental health, Nature, Well-being

## Abstract

**Background:**

Gardening and horticultural therapy (HT) has been widely recognised as a multicomponent approach that has affected a broad range of health and well-being outcomes. The aim of this umbrella review and meta-analysis was to compare the findings of previous reviews on the impact of multiple gardening interventions and gardening attributes on different well-being constructs.

**Methods:**

Electronic databases including PubMed, Web of Science, Science Direct, the Cochrane Library, and Google Scholar were searched from inception to December 2022. Interventional and observational reviews were eligible for inclusion in this umbrella review. Outcome measures included mental well-being, health status and quality of life. The key exposure variables were gardening and horticultural therapy. Narrative synthesis was used to evaluate the overall impact of gardening and HT on study outcomes. For a subsample of studies with available quantitative data, a random effect meta-analysis was conducted.

**Results:**

This umbrella review included 40 studies (10 interventional studies, 2 observational studies, and 28 mixed interventional and observational studies). The reviewed studies reported an overall positive impact of gardening activities on several measures of mental well-being, quality of life, and health status. Meta-analysis showed a significant and positive effect of gardening and HT activities on well-being (effect size (ES) 0.55, 95% confidence interval (CI) 0.23, 0.87, *p* < 0.001).

**Conclusions:**

Evidence from observational and interventional studies supports a positive role for gardening and HT activities on well-being and general health. Interventional studies with horticultural-based therapies were effective in improving well-being and quality of life both in the general population and vulnerable subgroups. The high degree of heterogeneity in the included studies cautions against any direct clinical implications of the study findings.

**Supplementary Information:**

The online version contains supplementary material available at 10.1186/s13643-024-02457-9.

## Background

Well-being, encompassing constructs such as positive affect, purpose, and life satisfaction, represents an important patient-centred outcome associated with multiple health benefits. Several studies, for instance, have linked higher levels of well-being with reduced risk of chronic diseases, improved immune functioning, fast recovery, and increased longevity [1–6]. However, previous literature attempting to summarise existing reviews on this topic, have narrowly focused on specific gardening activity or single well-being constructs including type of gardening, mental health status, or quality of life. There is however a need to evaluate multiple gardening activities and well-being outcomes examined in previous systematic reviews and meta-analyses, to enable for novel comparative insights. Therefore, our umbrella review based on existing systematic reviews and meta-analyses aimed to synthesise the state of knowledge on gardening-centred activities’ impact on multiple well-being outcomes and evaluate the quality of the reviewed evidence.

Increasingly, access to green spaces has meaningful therapeutic applications, especially for people with mental health conditions [7]. These therapeutic applications also extend to those with physical conditions who might benefit from the physical activity side, but also might experience emotional and cognitive benefits [8]. The positive aspect of gardening is that it efficiently combines physical with recreational activities, impacting on emotional, physical, and social well-being. Several systematic reviews of clinical trials and observational studies have documented multiple therapeutic benefits of gardening interventions across diverse populations and life domains [9–12]. Despite the growing number of systematic reviews on the topic, our understanding of the overall effect of gardening activities on different well-being constructs remains inconclusive.

Umbrella reviews enable a fast and effective understanding of the overall quality of evidence on a broad but well-defined topic (such as well-being) by integrating data from previous systematic reviews [13, 14]. The current study describes the results of an umbrella review which aimed to assess the quality of evidence from previous reviews on the impact of multiple gardening interventions and gardening attributes on different well-being constructs. This approach was considered necessary given that previous reviews incorporated evidence distributed across the globe, evaluating different aspects of gardening and well-being constructs. In this sense, we have aimed to contrast and compare the findings of published systematic reviews over the past two decades on the impact of gardening on well-being and related constructs.

## Methods

This umbrella review followed the Preferred Reporting Items for Systematic Reviews and Meta-Analysis (PRISMA) guidelines. The key questions of the review were:

1. To what extent do gardening and horticultural interventions reduce the risk of poor well-being, mental health, and quality of life?
2. Do the benefits of gardening and horticultural interventions depend on timing, intensity, or duration of activities?

### Search strategy and selection criteria

We systematically searched PubMed, Cochrane Library, Scopus, Science Direct Freedom Collection, Elsevier, and Web of Science to identify systematic reviews and meta-analyses that reported the impact of gardening on mental health and/or well-being among adults. We also ran similar queries on Google Scholar in December 2022 to identify systematic reviews or meta-analyses that might have previously been missed, and we examined the first 50 hits from each combination. We relied on MESH terms to identify all the relevant keywords for the search strategy (Supplementary Table S1), e.g. (“reviews” OR meta-analyses*) AND (garden* OR hortic*) AND (well* OR benef*).

Two investigators (IP, RMS) independently retrieved and assessed the full text of potentially eligible articles. The search was restricted to studies that included reviews on qualitative and quantitative studies, including systematic reviews, scoping reviews, rapid reviews, meta-analyses, and other types of reviews. We excluded reviews of research on school gardens; reviews that report research on passive use of gardens (walking, sitting, etc.); duplicate publications, abstracts or posters from conferences, and other summaries; reviews that include theoretical studies or text and opinion as their primary source of evidence; reviews published in foreign languages with no provision of English translation. The literature search was updated to the 30th of January 2023.

Reviews were included if described findings on the association between gardening and well-being in adults over the age of eighteen. To be inclusive, we considered all reviews that included different population groups, including people independently living, those living in residential or care homes, as well as specific clinical populations (e.g. dementia and Alzheimer).

The exposure of interest was different types of gardening, including home-gardening, allotment or community gardening, and therapeutic gardening. Furthermore, the attributes of gardening encompass frequency, duration, and intensity. We define gardening to include domestic, recreational, and therapeutic activities with gardening as a key component. The outcomes of interest were measures of well-being, mental health, and quality of life. Furthermore, published systematic reviews with or without meta-analysis of quantitative or qualitative studies were eligible. No limitations were placed on the design of the studies included in the reviews.

### Data extraction and quality assessment

Two independent researchers extracted the data (IP, RMS), and in the case of discrepancies, consensus was reached. From each eligible article, we extracted the first author’s name, year of publication, databases searched, country of study, total sample size, well-being measures, and the number of primary studies. For the meta-analysis, we also extracted (where available) the study-specific risk estimates (standardised mean differences, odds ratio, and relative risk) along with their 95% confidence intervals (CI).

Two of the authors (PI and AD) independently conducted Quality assessments of eligible studies using A MeaSurement Tool to Assess Systematic Reviews 2 (AMSTAR 2) — a critical appraisal tool for systematic reviews that include randomised or non-randomised studies. The instrument includes 16 items and 7 critical domains: protocol registered before commencement of the review; adequacy of the literature search; justification for excluding individual studies; risk of bias from individual studies being included in the review; appropriateness of meta-analytical methods; consideration of risk of bias when interpreting the results of the review; assessment of presence and likely impact of publication bias. We have used the online checklist for rating overall confidence in the results of the reviews (high, moderate, low, and critically low) [15]. Any discrepancies were resolved by discussion and consensus. Due to the low number of studies identified for this review, we have not excluded low-quality or critically low-quality studies from the review.

### Statistical analysis

We used random-effects models to estimate the summary effect size and associated 95% CI for each meta-analysis [16]. We used the prediction interval (PI) to evaluate the uncertainty for the effect size that would be expected in a new study estimating the same association [17]. Due to the heterogeneity in estimation measures (e.g. mean difference, standardised mean difference), we conducted subgroup analysis by estimation measure. To quantify the between-study heterogeneity we used the I^2^ metric that quantifies the variability in effect estimates that is due to heterogeneity rather than sampling error [18]. Values of *I*^2^ exceeding 50% or 75% denote large or very large heterogeneity, respectively. This approach ensured that all results from each meta-analysis were considered to assess the epidemiological credibility of the observed associations. Associations with a statistically significant effect of *P* < 10^−6^ and large sample size (> 1000 participants) and *I*^2^ < 50% (low heterogeneity) were deemed as providing the strongest level of epidemiological credibility. All statistical analyses were implemented using Stata V.17 (College Station Texas, US).

## Results

### Identification of eligible systematic reviews

**Fig. 1:**
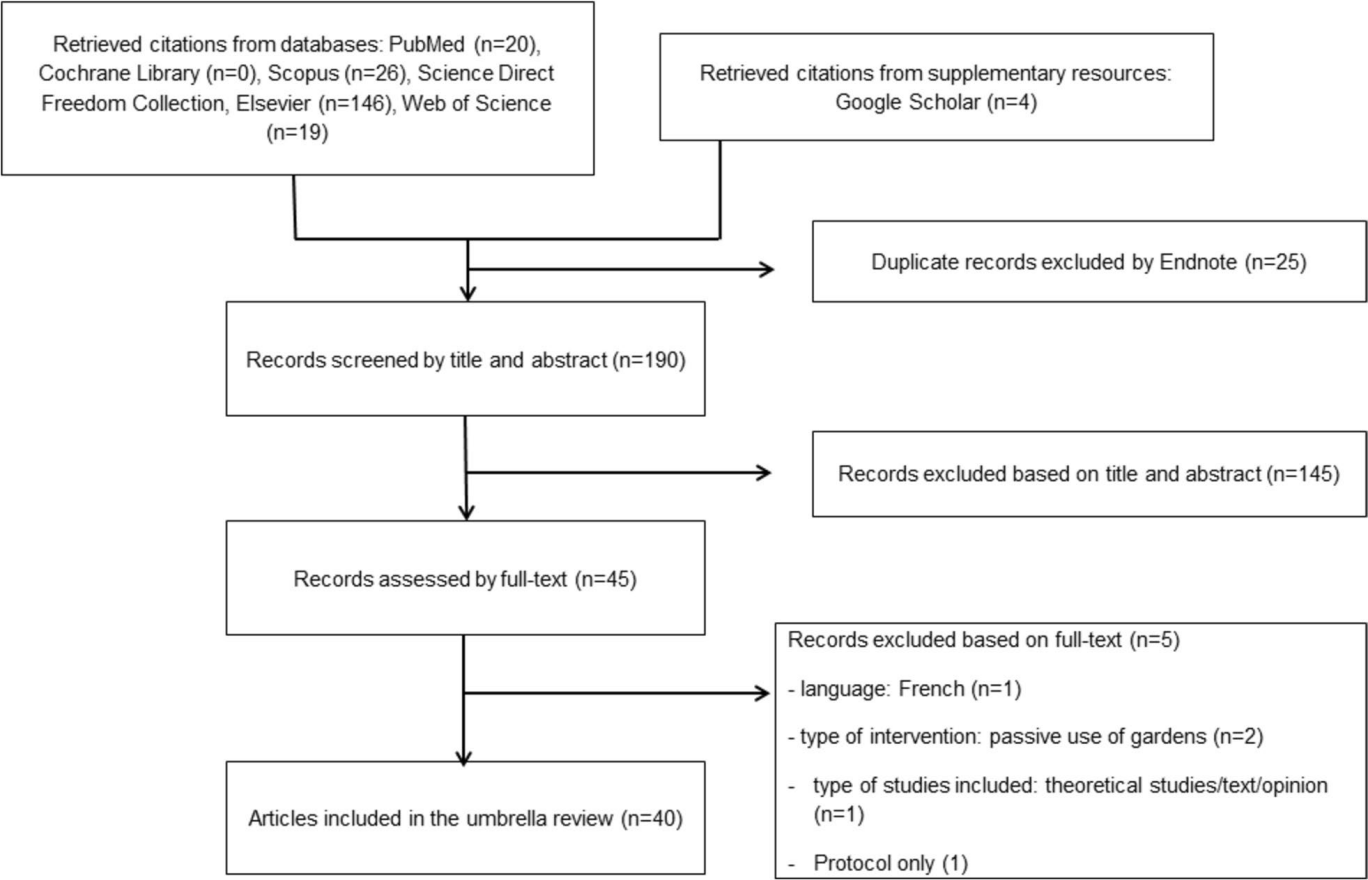
Flow chart illustrating the selection of systematic reviews

A total of 190 papers published between 2000 and 2022 were initially retrieved from database searching. After screening each paper by title and abstract, 45 papers remained to assess for eligibility through full-text review. In the end, we have included 40 papers that meet the set eligibility criteria in this umbrella review (Fig. 1 describes the process for identifying eligible studies and reasons for exclusion). The selective characteristics of the included papers using an interventional study design are included in Table 1 (see Supplementary Table S2 for a full list of included studies).

**Table 1:** Characteristics of the included interventional-based studies — a full list of included studies is included as Supplementary material (Table S2)

Author/Year	Number of studies (sample)	Country	Study design	Exposure	Outcome measures	Key findings
**Briggs et al. (2023) **[19]	20 (*N* = 874)	UK	Interventional	Horticultural therapy	Well-being, mental health, quality of life	+ impact on well-being; ? impact on mental health or quality of life
**Coventry et al. (2021) **[9]	50 (*N* = 4238)	UK	Interventional	Gardening	Mental health	+ impact on mental health
**Gregis et al. (2021) **[20]	84 (NA)	Italy	Interventional	Gardening; horticultural therapy	Well-being, mental health	? impact on well-being and mental health
**Kamioka et al. (2014) **[21]	4 (*N* = 248)	Japan	Interventional	Horticultural therapy	Well-being, mental health, quality of life	+ impact on mental health and behaviour
**Kondo et al. (2018) **[22]	43 (*N* = 1915)	USA	Interventional	Gardening	Mental health	+ impact on cortisol; ? impact for mood, anxiety
**Lin et al. (2021) **[23]	10 (*N* = 884)	China	Interventional	Horticultural therapy	Well-being, mental health, quality of life	+ impact on mental health, quality of life, and well-being
**Lin et al. (2022) **[24]	16 (*N* = 960)	UK	Interventional	Horticultural therapy	Well-being	+ impact on well-being
**Spano et al. (2020) **[25]	7 (*N* = 1699)	Italy	Interventional	Gardening; horticultural therapy	Well-being	+ impact on well-being
**Tu and Chiu (2020) **[26]	10 (*N* = 340)	Taiwan	Interventional	Horticultural therapy	Mental health	+ impact on mental health (cognition)
**Zhang et al. (2022) **[27]	13 (*N* = 687)	China	Interventional	Horticultural therapy	Mental health — depression	+ impact on mental health

+ denotes positive effects; ? denotes unclear effects; − denotes negative effects

*NA* not available

The included papers reported on the association between different gardening activities with several outcomes, including well-being, quality of life (QoL), and mental health conditions (depression, anxiety). In terms of study designs, ten reviews included evidence from interventional studies, two from observational studies, and twenty-eight included both interventional and observational studies. Most papers included systematic reviews (*n* = 17), followed by systematic reviews and meta-analysis (*n* = 6), scoping reviews (*n* = 6), literature reviews (*n* = 6), meta-analysis (*n* = 3) and critical reviews (*n* = 2). Among the eligible papers, the majority (*n* = 28) included studies from across the globe, followed by Europe and the UK (*n* = 6). The country-of-origin data were unavailable for six studies.

### Quality of included studies

The included papers covered different attributes of gardening activity, including type, duration/timing, frequency, or intensity (Table 2). However, most papers defined gardening in general, without reference to specific attributes. Substantial heterogeneity existed in the definition of outcome measure, with studies covering diverse dimensions of well-being (e.g. psychological, cognitive, physical, and emotional), quality of life (e.g. social, individual) and mental health (e.g. depression, stress, anxiety, general health). Two reviews (5%) were rated as high quality, five reviews (12%) were rated as moderate quality, five reviews (12%) were rated as low in quality, and the remaining 29 (71%) were rated as critically low quality. Some of the key challenges related to the incomplete or lack of description about the rationale for selection of study designs (*n* = 20), no evaluation of the potential risk of bias in individual studies that were included in review (*n* = 19), no justification for the exclusion of studies (*n* = 29), and no provision of a satisfactory explanation for observed heterogeneity in results (*n* = 23).

**Table 2:** Quality assessment of the review studies considered for inclusion of systematic reviews

Author (year of publication)	Quality level			
	**Critically low**	**Low**	**Moderate**	**High**
Al-Delaimy and Web (2017) [28]	X			
Briggs et al. (2023) [19]			X	
Clatworthy et al. (2013) [29]	X			
Coventry et al. (2021) [9]		X		
Cruz-Piedrahita et al. (2020) [30]	X			
Dyg et al. (2020) [31]	X			
Egli et al. (2016) [32]	X			
Gagliardi and Piccinini (2019) [33]	X			
Galhena et al. (2013) [34]	X			
Genter et al. (2015) [35]	X			
Gonzalez and Kierkevold (2014) [36]	X			
Gregis et al. (2021) [20]	X			
Herod et al. (2022) [37]	X			
Howarth et al. (2020) [38]	X			
Kamioka et al. (2014) [21]			X	
Kondo et al. (2018) [22]	X			
Kunpeuk et al. (2020) [39]				X
Lakhani et al. (2019) [40]		X		
Lampert et al. (2021) [41]	X			
Lin et al. (2021) [23]	X			
Lin et al. (2022) [24]	X			
Lu et al. (2020) [42]		X		
Mmako et al. (2020) [43]	X			
Moeller et al. (2018) [44]	X			
Murray et al. (2019) [10]			X	
Nicholas et al. (2019) [45]	X			
Poulsen et al. (2015) [11]	X			
Scott et al. (2022) [46]			X	
Soderback et al. (2004) [47]	X			
Soga et al. (2017) [48]		X		
Spano et al. (2020) [25]	X			
Tharrey and Darmon (2021) [49]	X			
Tu and Chiu (2020) [26]	X			
Uwajeh et al. (2019) [50]	X			
Wang et al. (2013) [51]	X			
Wang et al. (2022) [12]				X
Whear et al. (2014) [52]			X	
York and Wiseman (2012) [53]	X			
Zhang et al. (2021) [54]	X			
Zhang et al. (2022) [27]		X		

#### Associations of gardening with well-being, mental health, and quality of life outcomes

##### Well-being

Overall, the included reviews (Table 1) documented a positive effect of gardening and/or horticultural therapy on multiple measures of psychological well-being (e.g. neighbourhood cohesion, trust, and social networking) and physiological well-being. All gardening activities and interventions appeared to provide benefits to psychological and/or physiological well-being [25, 32, 35, 37, 39]. Reviews that focused on specific populations also documented that horticulture-based therapy had a positive impact on the mental and physical well-being of people with dementia [46, 52], mental disorders [19, 50], those with physical long-term conditions as well as older populations [11, 24, 38, 47].

##### Mental health

The included reviews that examined health status were unanimous in documenting a positive impact of gardening activities on a range of mental health outcomes, such as depression and anxiety symptoms, stress, mood disturbance, and cognitive function [20, 22, 30, 36, 48, 49, 53]. The review by Coventry et al. [9] reported a beneficial role of gardening in reducing symptoms of anxiety and negative effects in people with several mental illnesses. Horticultural therapy was also found to be effective in reducing depressive symptoms and improving cognitive performance [10, 21, 26, 27, 33, 40, 42, 50, 54].

##### Quality of life

Regarding quality of life (QoL) outcomes, standard indicators of QoL included life satisfaction, social safety, security, and freedom. The majority of the included reviews reported improvements in different aspects of quality of life (social relations/connections, independent living, and health status) related to horticultural therapy [37, 44, 45] and gardening [28, 34, 41]. Community gardens also had beneficial effects on QoL (e.g. personal control, self-esteem, social connections) both among vulnerable [12, 23, 29, 31, 43, 46, 51] and general populations [41, 48].

##### Meta-analysis results

**Fig. 2:**
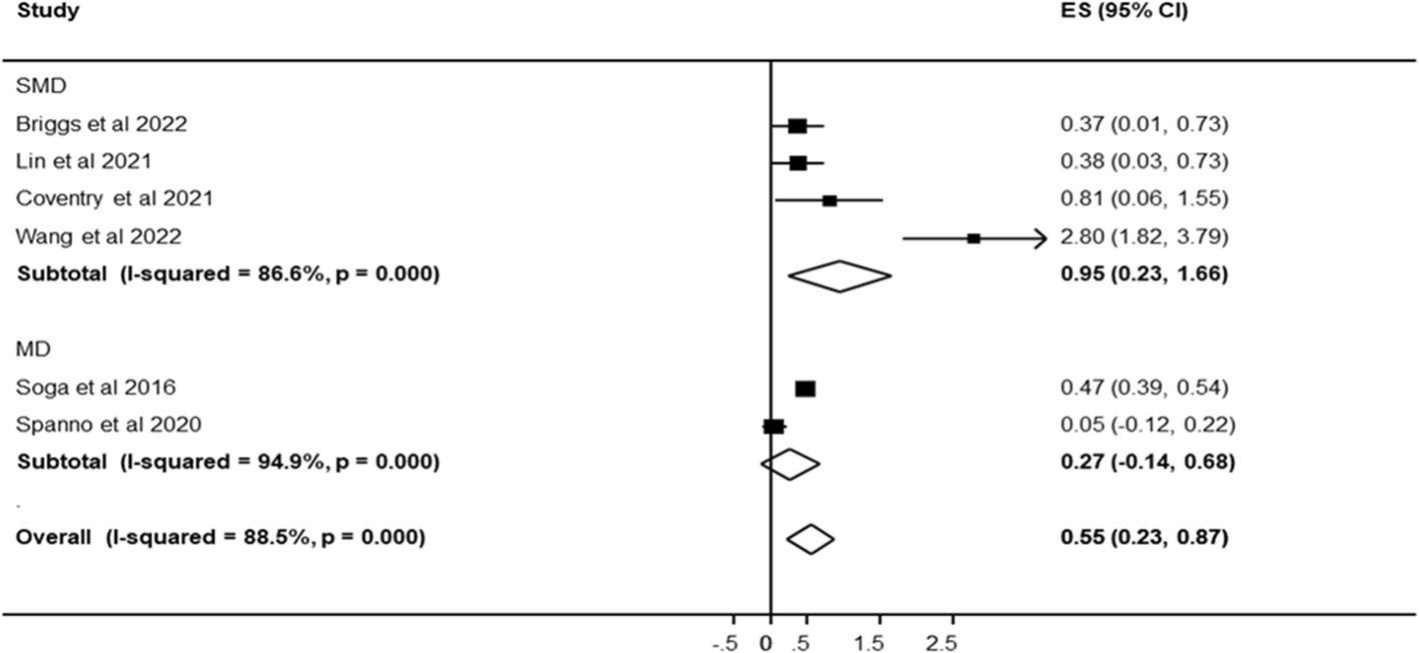
Effects of gardening and horticultural interventions on well-being and mental health

A random effects meta-analysis (Fig. 2) on a subsample of the included studies with available data [9, 12, 19, 23, 25, 48], indicated an overall 55% increment in well-being measures (95% CI: 0.23–0.87) for gardening. This interpretation is made cautiously given the variation in measures of effect size (e.g. smd vs md) between the studies. Heterogeneity across the included studies was statistically significant (*I*^2^ = 88.5%, *p* < 0.001).

## Discussion

This umbrella review presented a comprehensive overview of several gardening activities and interventions on diverse well-being outcomes reported in 40 systematic reviews, including 6 meta-analyses, based on research covering over two decades and appraised the quality of evidence. The study findings should be interpreted cautiously given the substantial heterogeneity between available meta-analyses and low quality of the majority of the included reviews. As a result, current evidence does not allow strong recommendations about the benefits of gardening activities and horticultural interventions for well-being outcomes. The low quality, as assessed in this review, of published evidence means that the likely true effects of gardening and horticultural interventions for well-being might be different from the best estimates [55]. To address this concern, future studies should follow recommended reporting guidelines in order to facilitate critical appraisal of the evidence and enhance the validity and reliability of published findings. The journals have a key role in this respect. The findings of the current study are of value nevertheless by documenting the current state of evidence about the benefits of gardening activities and horticultural interventions for population well-being. By highlighting critical gaps in current literature, it offers important avenues for future research concerning the effectiveness of gardening interventions for improving population well-being.

Multiple biopsychosocial factors may account for a direct or indirect positive impact of gardening activities on well-being, quality of life and health status. For some people, gardening activities lead to adoption of healthy behavioural practices (e.g. increased fruit and vegetable consumption, and physical activity) that positively impact on several aspects of health and well-being [56–58]. Proximity to nature, because of gardening, infuses feelings of connectedness with nature that promotes positive affect, lifted mood, and tranquillity. Spending time outdoors in a relaxed atmosphere can make people more mindful of the present, gain emotional resilience, and combat stress through greater vitality [59, 60]. Several studies have documented that spending time in nature triggers physiological responses that lower stress levels. According to the attention restoration theory, connectedness with nature replenishes cognitive resources, leading to improved concentration and attention [61]. Community gardening also provides a safe and relaxed context for social interaction, which can counteract feelings of loneliness and social isolation, particularly among vulnerable groups such as people with pre-existing learning difficulties and mental health [57]. It provides an opportunity for greater community cohesion and social connectedness, increasing one’s network of social support. Further, gardening activities have direct physiological benefits in terms of reduced blood pressure and obesity levels, thus reducing the risk of physical health disorders (e.g. vascular diseases, type 2 diabetes, and cancer) [62].

In our review, we have identified that some published systematic reviews suffered from methodological flaws in the identification of eligible studies. Much of the research of Cruz-Piedrahitra et al. [30] was based on self-reported behaviours or assessments; from a total of 138 studies included, only five were longitudinal studies. To conduct a meta-ethnography, York and Wiseman [53] included only qualitative studies making it difficult to judge what the authors chose to edit from the original findings and discussion, and whether this would have altered the research findings. Gonzalez and Kirkevold [36] specified that the small sample sizes and the lack of randomised controlled studies were too difficult to establish causal relationships.

A key concern related to our study findings is around causality. Firstly, gardening covers a wide range of activities and influences. If communal gardening or working close to ‘nature’ have an impact on well-being it might be through multiple pathways, not all related to gardening per se. Secondly, gardening might not have a direct influence on well-being but rather encourages exercise and healthy diet, with well-being being a consequence of these behaviours. Thirdly, it is often challenging to ascertain the direction of association; which comes first, gardening or physical activity and well-being? It might be that people who are engaged in gardening activities are more physically fit or have high levels of well-being. Even in interventional studies, participants who are likely to benefit should have a certain degree of fitness beforehand.

### Limitations and strengths

A notable strength of our study is the integration of quantitative evidence from different gardening-based interventions on multiple well-being-centred outcomes. Integration of a larger scale of evidence helps to better understand the strengths and limitations of the current data guiding public health initiatives aimed to improve population well-being [63].

Our umbrella review only considered for inclusion the highest level of evidence, namely systematic reviews, and meta-analysis. The reviews comprised different study designs and not all of them measured the same outcomes. The definition of gardening also varied across studies, making it difficult to integrate findings. Reviews that included cross-sectional studies are limited by the fact that causality cannot be established. Unfortunately, most of the reviews available to us did not present data on gardening and health and well-being separately by type of study design, impeding us from making a robust assessment of causality or direction of association between gardening and improved quality of life, well-being, and health outcomes. Future reviews which include only prospective or longitudinal designs are needed to enable any causal inference. However, the small number of reviews that focused on clinical trials found that gardening has a positive impact on health and well-being. Meta-analyses of randomised clinical trials would provide the strongest level of evidence for the effectiveness of gardening on well-being and health status. Another limitation of the current umbrella review is that the quality of the majority of included reviews was judged to be critically low-quality according to the AMSTAR 2 criteria. Combining low-quality studies with high-quality studies could lead to erroneous conclusions if the quality of studies is ignored [64]. Our study used an established tool for detailed analysis of the quality of available evidence which should facilitate progress within the horticultural therapy field. Furthermore, excluding critically low studies from the meta-analysis has resulted in a higher overall effect size, suggesting that our analysis has underestimated the true effect of horticultural interventions on well-being.

### Conclusions

To conclude, this umbrella review identified a positive association between gardening and horticultural therapy and multiple measures of well-being, quality of life, and health status. Existing reviews did not provide more granular evidence in terms of different aspects of gardening (e.g. type, quantity, and intensity), and therefore this should be a priority for future studies. Several biopsychosocial and physiological mechanisms potentially account for the observed associations. Methodologically robust randomised controlled studies are needed, however, to test causal associations between specific gardening activities and well-being. Similarly, evidence-based information is needed on how to facilitate and support greater engagement with community gardens and nature for inner urban populations that present with the highest prevalence of mental and physical long-term conditions.

### Supplementary Information

**Additional file 1: Table S1.** Search strategy for the eligible systematic reviews. **Table S2.** Comprehensive description of the included systematic reviews.

## Data Availability

Data sharing is not applicable to this article as no datasets were generated or analysed during the current study.
