# Supplementary material for: The impact of gardening on well-being, mental health, and quality of life: an umbrella review and meta-analysis
Source: Syst Rev. 2024 Jan 29;13:45. doi: 10.1186/s13643-024-02457-9 (PMC10823662; doi:10.1186/s13643-024-02457-9)
Supplement: Supplementary file 1 — Additional file 1: Table S1. Search strategy for the eligible systematic reviews. Table S2. Comprehensive description of the included systematic reviews. [file 13643_2024_2457_MOESM1_ESM.docx]

Table S1. Search strategy for the eligible systematic reviews

| **No** | **Database** | **Search Terms** | **Filters** |
| --- | --- | --- | --- |
| **1** | PubMed | ((garden*[Title]) OR (hortic*[Title])) AND ((benef*[Title]) OR (well*[Title])) | Meta-Analysis, Review, Systematic Review, English |
| **2** | Scopus | ((garden*[Title]) OR (hortic*[Title])) AND ((benef*[Title]) OR (well*[Title])) | Review, English |
| **3** | Web of Science | (((TI=(garden*)) OR TI=(hortic*)) AND ((TI=(well*)) OR TI=(benef*))) | Review Article, English |
| **4** | Science Direct Freedom Collection, Elsevier | gardening or horticulture and benefits or well-being | Review articles |
| **5** | Google Scholar | gardening benefits and well-being review, meta-analysis; horticulture benefits and well-being review, meta-analysis; gardening and horticulture benefits review | Meta-analysis, Review article |
| **6** | Cochrane Library | gardening and wellbeing, gardening and benefits, horticulture and benefits, horticulture and wellbeing | Review |

Table S2. Comprehensive description of the included systematic reviews

| **Author/year** | **Type of study** | **Number of primary studies (total sample size)** | **Period covered** | **Database searched** | **Country of study** | **Study design** | **Gardening factors** | **Outcome measures** | **Relevant findings** |
| --- | --- | --- | --- | --- | --- | --- | --- | --- | --- |
| Al-Delaimy and Web (2017) | Literature review | 37 (N=10491) | 1991-2015 | PubMed | USA | Mixed | Gardening | Health status | + impact on mental health |
| Briggs et al. (2022) | Systematic review and meta-analysis | 20 (N=874) | 2010-2021 | PubMed | UK | Interventional | Horticultural therapy | Well-being; mental health – stress; quality of life | + impact on well-being;  ? impact on mental health or quality of life |
| Clatworthy et al. (2013) | Critical review | 10 (N=314) | 2004-2011 | Science Direct | UK | Mixed | Horticultural therapy | Mental health – anxiety, depression, mood | + impact on mental health |
| Coventry et al. (2021) | Systematic review and meta-analysis | 50 (N=4238) | 2010-2021 | Science Direct | UK | Interventional | Gardening | Mental health – affect, stress, anxiety, depression | + impact on mental health |
| Cruz-Piedrahita et al. (2020) | Scoping review | 138 (NA) | 2005-2018 | Science Direct | UK | Mixed | Gardening | Well-being; general health status – anthropometric measurements; mental health – anxiety, stress, depression; social cohesion | + impact on mental health, and quality of life |
| Dyg et al. (2019) | Literature review | 51 (N=26760) | 2000-2017 | PubMed | Denmark | Mixed | Horticultural therapy | General health status – anthropometric measurements; mental health – stress, depression; social health; quality of life | + impact on health and quality of life |
| Egli et al. (2016) | Literature review | 12 (NA) | 2004-2015 | PubMed | New Zealand | Mixed | Gardening | Well-being; health status | + impact on well-being and mental health |
| Gagliardi and Piccinini (2019) | Literature review | 42 (N=28489) | 2005-2018 | Science Direct | Italy | Mixed | Gardening; Horticultural therapy | Well-being; general and mental health status – behaviour, sleep, stress, depression, mood, cognitive function | + impact on mental health and well-being |
| Galhena et al. (2019) | Systematic review | NA | NA | Scopus | USA | Mixed | Gardening | Well-being | + impact on well-being and quality of life |
| Genter et al. (2015) | Systematic review | 10 (N=412) | 1999-2013 | Scopus | UK | Mixed | Gardening | Well-being; health status | + impact on health and well-being |
| Gonzales and Kierkevold (2013) | Scoping review | 16 (N=643) | 1997-2010 | PubMed | Norway | Mixed | Horticultural therapy | Mental health – sleep, affect, cognitive function | + impact on mental health |
| Gregis et al. (2021) | Systematic review | 84 (NA) | NA | PubMed | Italy | Interventional | Gardening; Horticultural therapy | Well-being; general and mental health | ? impact on well-being and mental health |
| Herod et al. (2022) | Systematic review | 35 (N=5971) | 2010-2021 | Scopus | Poland | Mixed | Horticultural therapy | Well-being; health status; quality of life | + impact on mental health and well-being |
| Howarth et al. (2020) | Scoping review | 77 (NA) | 2000-2020 | PubMed | UK | Mixed | Horticultural therapy | Well-being; health status; quality of life | + impact on health and well-being |
| Kamioka et al. (2014) | Systematic review | 4 (N=248) | 2003-2010 | PubMed | Japan | Interventional | Horticultural therapy | Well-being; general and mental health – affect, stress, anxiety, depression; quality of life | + impact on mental health and behaviour |
| Kondo et al. (2018) | Systematic review | 43 (N=1915) | 1991-2016 | Science Direct | USA | Interventional | Gardening | Mental health – stress, mood, anxiety | + impact on cortisol; ? impact for mood, anxiety |
| Kunpeuk et al. (2019) | Systematic review and meta-analysis | 19 (NA) | 1991-2017 | Google Scholar | Thailand | Mixed | Gardening | Health status | + impact on health status (behaviour) |
| Lakhani et al. (2019) | Systematic review | 18 (NA) | 1996-2017 | Science Direct | Australia | Mixed | Horticultural therapy | Mental/emotional and social health | + impact on mental health |
| Lampert et al. (2021) | Systematic review | 8 (N=107) | 1991-2020 | PubMed | Portugal | Observational | Gardening | Well-being; general health status – BMI; mental health – anxiety, stress, depression; life satisfaction; quality of life | + impact on mental health and well-being |
| Lin et al. (2021) | Systematic review and meta-analysis | 10 (N=884) | 2011-2020 | PubMed | China | Interventional | Horticultural therapy | Well-being; general and mental health – agitation, anxiety, stress, depression; life satisfaction; quality of life | + impact on mental health, quality of life, and well-being |
| Lin et al. (2022) | Systematic review | 16 (N=960) | 2006-2021 | Scopus | UK | Interventional | Horticultural therapy | Well-being | + impact on well-being |
| Lu et al. (2019) | Systematic review and meta-analysis | 23 (N=938) | 1992-2018 | Google Scholar | China | Mixed | Horticultural therapy | Mental health – behaviour | + impact on well-being and quality of life |
| Mmako et al. (2020) | Scoping review | 19 (N=850) | 2009-2019 | Science Direct | Australia | Mixed | Horticultural therapy | Well-being; mental health – stress, cognitive function; social health; quality of life | ? impact on mental health and quality of life |
| Moeller et al. (2018) | Scoping review | 85 (NA) | 1986-2016 | PubMed | UK | Mixed | Horticultural therapy | General and mental health – stress; quality of life | - impact on quality of life |
| Murray et al. (2019) | Systematic review | 31 (N=341) | 1993-2017 | Web of Science | UK | Mixed | Horticultural therapy | Mental health – affect, anxiety, depression; quality of life | + impact on quality of life, well-being and mental health |
| Nicholas et al. (2019) | Systematic review | 20 (N=684) | 2008-2018 | Science Direct | Singapore | Mixed | Horticultural therapy | Well-being; general (anthropometric measurements) and mental health – agitation, affect, anxiety, stress, depression; quality of life | + impact on mental health, well-being |
| Poulsen et al. (2015) | Systematic review | 19 (N=422) | 1992-2013 | Science Direct | Denmark | Mixed | Horticultural therapy | Well-being; mental health – anxiety, stress | + impact on well-being |
| Scott et al. (2022) | Systematic review | 8 (N=178) | 2002-2021 | PubMed | Australia | Mixed | Horticultural therapy | Well-being | + impact on well-being, mental health |
| Soderback et al. (2004) | Systematic review | 68 (NA) | 1957-2003 | PubMed | Sweden | Mixed | Horticultural therapy | Well-being; general and mental health – cognitive function; life satisfaction | + impact on mental health and well-being |
| Soga et al. (2016) | Meta-analysis | 22 (N=2578) | 2002-2016 | PubMed | UK | Mixed | Gardening; Horticultural therapy | Well-being; health status; quality of life | + impact on well-being |
| Spano et al. (2020) | Meta-analysis | 7 (N=1699) | 2010-2017 | Scopus | Italy | Interventional | Gardening; Horticultural therapy | Well-being | + impact on mental health and well-being |
| Tharrey and Darmon (2021) | Systematic review | 15 (N=18473) | 2008-2020 | PubMed | France | Mixed | Horticultural therapy | Well-being; general and mental health; life satisfaction; quality of life | + impact on mental health (cognition) |
| Tu and Chiu (2020) | Meta-analysis | 10 (N=340) | 2006-2019 | Google Scholar | Taiwan | Interventional | Horticultural therapy | Cognitive function | + impact on mental health (cognition) |
| Uwajeh et al. (2019) | Literature review | 29 (NA) | NA | Science Direct | Turkey | Mixed | Horticultural therapy | General and mental health – stress, depression, cognitive function; quality of life | + impact on mental health and well-being |
| Wang et al. (2013) | Systematic review | 22 (N=2300) | 1979-2010 | PubMed | USA | Mixed | Gardening; Horticultural therapy | Well-being; health status; quality of life | + impact on quality of life and mental health |
| Wang et al. (2022) | Systematic review and meta-analysis | 15 (N=1046) | 2006-2018 | PubMed | China | Mixed | Horticultural therapy | Health status | + impact on mental health |
| Whear et al. (2014) | Systematic review | 17 (N=426) | 1992-2012 | PubMed | UK | Mixed | Gardening; Horticultural therapy | Physical and mental well-being | + impact on well-being and mental health |
| York and Wiseman (2012) | Critical review | 4 (N=172) | 2003-2007 | Web of Science | UK | Observational | Gardening; Horticultural therapy | Well-being; health status | + impact on mental health |
| Zhang et al. (2021) | Scoping review | 54 (NA) | 1991-2021 | PubMed | China | Mixed | Gardening | Mental health – affect, depression | + impact on mental health |
| Zhang et al. (2022) | Systematic review | 13 (N=687) | 2013-2021 | Google Scholar | China | Interventional | Horticultural therapy | Mental health – depression | + impact on depressive symptoms |

Note: + denotes positive effects; ? denotes unclear effects; - denotes negative effects; NA – not available
